# Recent Advances in Development and Application of Physiologically-Based Pharmacokinetic (PBPK) Models: a Transition from Academic Curiosity to Regulatory Acceptance

**DOI:** 10.1007/s40495-016-0059-9

**Published:** 2016-04-14

**Authors:** Masoud Jamei

**Affiliations:** Simcyp Limited (a Certara Company), Blades Enterprise Centre, John Street, Sheffield, S2 4SU UK

**Keywords:** Physiologically-based pharmacokinetics, Systems pharmacology, In vitro in vivo extrapolation, Modelling and simulation, Regulatory science, Precision medicine

## Abstract

There is a renewed surge of interest in applications of physiologically-based pharmacokinetic (PBPK) models by the pharmaceutical industry and regulatory agencies. Developing PBPK models within a systems pharmacology context allows separation of the parameters pertaining to the animal or human body (the system) from that of the drug and the study design which is essential to develop generic drug-independent models used to extrapolate PK/PD properties in various healthy and patient populations. This has expanded the classical paradigm to a ‘predict-learn-confirm-apply’ concept. Recently, a number of drug labels are informed by simulation results generated using PBPK models. These cases show that either the simulations are used in lieu of conducting clinical studies or have informed the drug label that otherwise would have been silent in some specific situations. It will not be surprising to see applications of these models in implementing precision dosing at the point of care in the near future.

## Introduction

Physiologically-based pharmacokinetic (PBPK) models map drug movements in the body to a physiologically realistic compartmental structure using sets of differential equations. It is suggested [30] that the origins of PBPK models go back to the work of Teorell in 1937 [36]. Teorell appreciated that an integrated model is needed to account for various processes affecting drug disposition around the body. As computational power increased, PBPK models were further developed in the 1960s and the 1970s, and the first article which appeared with the term *PBPK* in its title is [11]. The majority of early applications of PBPK models deal with issues related to anaesthesia and risk assessment of environmental chemicals due to their capability to predict the systemic exposure of chemicals in various parts of the body [30].

Recently, there has been a renewed surge of interest in applications of PBPK models by the pharmaceutical industry, especially in populations where designing and conducting clinical studies is more challenging [17]. The trend is part of wider applications of modelling and simulation (M&S) in the industry. A recent survey focusing on preclinical pharmacokinetic/pharmacodynamics (PK/PD) analysis was conducted across pharmaceutical companies who are members of the International Consortium for Quality and Innovation (IQ) in Pharmaceutical Development [34]. Based on the survey responses, ∼68 % of companies use preclinical PK/PD analysis in all therapeutic areas indicating its broad application, and the majority (∼86 %) indicated that systems pharmacology models are ‘sometimes’ used.

Various factors have contributed to this rise in interest, including the increased cost of developing new drugs and progress made in better understanding the biology of systems making up the PBPK models and in particular the ability to predict enzyme and transporter functions in organs [27]. A recent study by Poggesi and co-workers stated that while, since 2000, there has been an almost exponential rise in the use of PBPK models in the field of drug research and development, the number of publications using PBPK models for non-pharmaceutical agents has been almost at a steady-state level [23]. Commercial software platforms that facilitate rapid deployment of PBPK models have contributed to the increased use of PBPK models. Further, they paved the way for non-modellers, who historically could not easily use such models, to utilise PBPK models. Software features and values and limitations of both the ‘ready to use’ and the traditional user customizable packages are reviewed and compared elsewhere [3].

In this review, recent advances in developing PBPK models and their applications, leveraging population pharmacokinetic (PopPK) techniques in improving PBPK model performance, the impact of these models on regulatory sciences and applications and future directions are briefly discussed.

### IVIVE-Linked PBPK Models in a Systems Pharmacology Context

By their nature, PBPK models are complex and depend on many parameters. Generally, these parameters represent combined effects of the administrated compound and the subject that the compound is administered to. For example, the fraction unbound in plasma (fu) is commonly considered as a drug parameter. However, in fact it is a combination of the drug affinity to human serum albumin and the individual’s albumin level in plasma [16]. PBPK models can be parametrised to either directly use fu, as a single value, or determine fu based on the individual’s albumin level and the drug affinity to albumin. The PBPK model structure in both of these approaches is the same. However, the latter approach allows integrating the body (system) and drug parameters to determine fu. Therefore, the covariates of PK properties, in this case the serum albumin level, are incorporated within the model which in turn facilitates predicting inter-subject variability [27].

In a systems pharmacology context, the PBPK model parameters should be divided into three categories, namely, the system or species (e.g. age, weight, height, genetic make-up, etc., of human or animal subjects), the drug (e.g. physicochemical characteristics determining permeability through membranes, partitioning to tissues, binding to plasma proteins, or affinities towards certain enzymes and transporter proteins) and the study design (e.g. dose, route and frequency of administration, the effect of concomitant drugs and food) [16]. This separation is vital to allow developing generic drug-independent models that can be used for a wide range of compounds. Further, it facilitates independent development of various databases of anatomical, biological, physiological and genetic characteristics of healthy and disease populations that can be used to simulate virtual clinical studies [27].

**Fig. 1:**
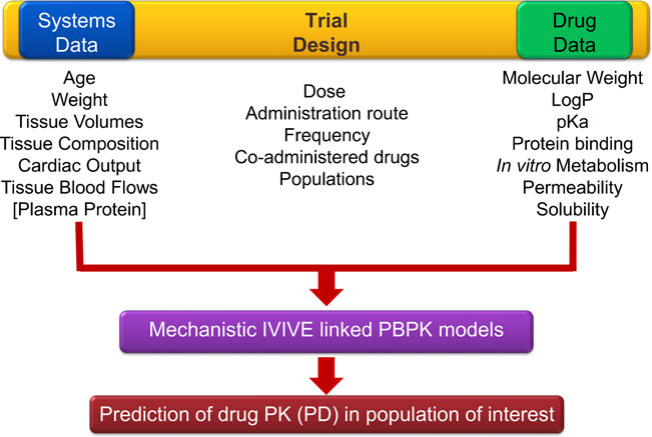
A schematic of systems pharmacology paradigm where the systems, drug and trial design data are mechanistically combined and integrated within PBPK models to simulate and predict the drug PK/PD in virtual populations. The Systems and Data are part of the trial setting thence included in the *Trial Design box*

The following factors have significantly expanded our ability to combine and integrate various prior datasets into PBPK models (see Fig. 1).

- The availability of in vitro systems which act as surrogates for in vivo reactions relevant to the absorption, distribution, metabolism and excretion (ADME) processes
- Recent development and refinement of in vitro–in vivo extrapolation (IVIVE) techniques [28];
- Methods to predict tissue partition coefficients using physicochemical properties and protein binding data [24, 25].

This systems approach expedites predicting and investigating intrinsic (e.g. organ dysfunction, race, genetics and disease) and extrinsic (e.g., drug–drug interactions, smoking, diet and environmental) factors on drug exposure and response that are required to be assessed during the drug development process [15]. This in turn helps with designing and optimising clinical studies and selecting the optimal dosing regimens [42]. Such capabilities become even more important when drugs are to be dosed in very young children or disease populations where running clinical studies is not commonly feasible or is very challenging [17].

### Bottom-Up and Top-Down: Complementary Paradigms

The systems pharmacology approach to PBPK modelling by its nature is a ‘bottom-up’ approach because it integrates many pieces of discrete information from different sources within a mechanistic framework. The ability to provide a priori estimates of inter-individual variability and identification of the characteristics of individuals at extreme risk are among the most important features of this approach, both of which are unique to population-based PBPK models. This bottom-up approach differs from the conventional way in which covariates affecting PK/PD behaviour are investigated. The latter, so called ‘top-down’, approach requires PK data from clinical studies in actual human (or animal) subjects which are analysed using different approaches including PopPK data analysis. An advantage of the bottom-up approach is that the simulation and prediction exercise can start at very early stage of drug development when the plasma concentration data are not yet available. Historically, Monte Carlo simulations are used when developing population PBPK models which commonly do not consider the parameter’s inter-dependencies. However, it is imperative to mechanistically incorporate covariates in these models and employ correlated Monte Carlo simulations instead [16]. In doing so, the predicted PK/PD properties are inherently affected by the relevant covariates.

After PBPK models are developed with the relevant parameters, they can be used to simulate and predict various ‘what-if’ scenarios, and when clinical data become available their ability in predicting real-world cases can be assessed. Given limitations in our knowledge and in some cases the paucity of necessary model parameters, predictions may not always accurately match the observed clinical data. On these occasions, the common PopPK data analysis techniques can be applied to improve the PBPK model performance and fine tune uncertain model parameters [31]. Generally, there is a larger degree of freedom in fitting PBPK model parameters since there are various parameters that can be fitted and provide similar outcomes, which can cause structural identifiability issues. To address this, among other methods, the use of global and/or local sensitivity analysis [21] and the Bayesian framework [19] are proposed. Tsamandouras and co-workers have discussed parameter estimation for PBPK models while highlighting the importance of considering the covariance structure between model parameters and the associated uncertainty and population variability [37].

In PopPK analysis model, performance and predictive capability are assessed commonly using various goodness-of-fit measures to make sure they are fit for purpose and that they are parsimonious (i.e. models contain as few estimated parameters as possible to accurately describe the dataset). While similar measures may be used to qualify system pharmacology models, there are cases where these measures are not applicable as argued by Agoram [2]. Specifically, the most parsimonious model is not always the most useful model for developing a mechanistic understanding of the drug disposition and/or action in subjects (system). Indeed, any discrepancy between the model predictions and observed data may itself be informative about an underlying mechanism that has not been adequately understood or incorporated. In the same vein, after fitting and fine tuning some of the PBPK model’s parameters, it is necessary to assess its performance for studies whose data were not used to fit the model parameters to verify the model [27].

In contrast to empirical/compartmental PKPD models, one of the main advantages of PBPK models is that they can be used to extrapolate outside the studied population and experimental conditions. This expands the classical paradigm to ‘predict-learn-confirm-apply’ [41, 42] that stretches the spectrum of M&S from early drug discovery to beyond phase III clinical studies.

### Applications of PBPK/PD Models

Applications of PBPK models go well beyond predicting PK properties, and they have been used in predicting and assessing drug efficacy and safety. PBPK models can provide estimation of local concentrations at the site of action and covariates that contribute to their inter-subject variability. Therefore, they often remove the need to use empirical effect compartments to connect plasma and effect concentrations. In a recent study, Rose and co-workers used a population-based PBPK model of rosuvastatin to investigate the *lack* of effect of genotype-dependent uptake by the organic anion-transporting polypeptide 1B1 (OATP1B1) transporter on the pharmacological response [26]. Observed data showed that the area under the plasma concentration-time curve (AUC0-infinity) was increased by 63 and 111 % for the c.521TC and c.521CC genotypes vs. the c.521TT genotype of the OATP1B1 transporter, while the PD response remained relatively unchanged (3.1 and 5.8 % reduction). Using local concentration at the effect site to drive the PD response enabled the PBPK model to explain the observed disconnection between the effect of the OATP1B1 polymorphism on the rosuvastatin plasma concentration and the cholesterol synthesis response while a classical PKPD model could not achieve this.

Chetty and colleagues reported four applications of integrating prior in vitro data and PBPK models with PD models [5]. In the first case, using the PBPK/PD model, they successfully predicted the impact of CYP2D6 phenotypic impact on the metoprolol PK and PD. In the second case, the PK and PD properties of a controlled release formulation of nifedipine were predicted using prior information and the immediate release formulation data. The operational model of agonism was used in the third application to describe the hypnotic effects of triazolam, and this was successfully extrapolated to zolpidem by changing only the drug-related parameters from in vitro experiments. And in the fourth case study, differences in QTc prolongation due to quinidine in Caucasian and Korean females were successfully predicted using free heart concentrations as an input to the PD models. The PBPK models can also be connected to quantitative system pharmacology models. Schaller and colleagues developed a generic PBPK model of the glucose–insulin–glucagon regulatory system for both healthy populations and type 1 diabetes subjects which features an insulin receptor model relating PK properties to PD effects [33].

Usually, the anatomical, biological, physiological and genetic data for healthy and Caucasian subjects are richer than for disease or ethnic populations. Combining bottom-up and top-down approaches can also assist with identifying/estimating unknown/uncertain systems parameters when developing new databases. In a recent study, Feng and co-workers proposed a general framework that PBPK modelling should be considered to predict ethnic sensitivity of PK properties prior to any human data and/or with data in only one ethnicity. They argued that PBPK modelling prediction and PopPK analysis confirmation can complement each other to assess ethnic differences in PK at different drug development stages [7].

### PBPK Impact on Regulatory Decisions

Over the last decade, PBPK modelling has had a significant impact on regulatory science and decisions. The US FDA has identified innovation in clinical evaluations (e.g. through M&S) as a major scientific priority area, and they have used M&S strategies to address various drug development, regulatory and therapeutic questions over the past decade [14]. Parekh and co-workers have highlighted some examples where M&S has served as a useful predictive tool which include dose selection for pivotal trials, dosing in select populations such as paediatrics, optimisation of dose and dosing regimen in a subset patient population, prediction of efficacy and dosing in an unstudied patient population, etc. [22].

### Distribution of Areas of PBPK Model Applications

**Fig. 2:**
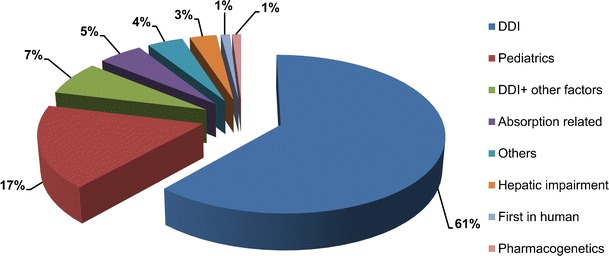
The distribution of area of applications of 136 regulatory submissions to FDA (until Oct 2014) where PBPK modelling has been applied, updated by Grillo [10] after [14]

Specifically, there has been a significant rise in the number of regulatory submissions to regulatory agencies that contain elements of PBPK modelling. Based on data presented by Grillo at the American Association of Pharmaceutical Scientists (AAPS) from 2008 until October 2014, there have been 136 FDA submissions using PBPK (Grillo 2014). Figure 2 shows what areas were covered by these submissions.

Looking at the publicly available data, Sager and co-workers collected data on the use of the PBPK models for pharmaceutical agents in humans published in English between 2008 and May 2015 where they found a total of 366 PBPK-related articles [32]. The picture of application areas was somehow different from that of the FDA submissions where drug–drug interaction (DDI)-related models cover only 28 %, followed by inter-individual variability and general clinical PK predictions 23 %, formulation or absorption modelling 12 % and predicting age-related changes in PK and disposition 10 %. When these new applications appear in regulatory submissions, we can expect the regulatory applications of PBPK models to be expanded and get closer to what is reported by Sager and co-workers.

It is not surprising that a big proportion of the applications are dealing with DDI and paediatrics. DDI is one of the very first areas tackled by IVIVE-linked PBPK models, and the recent regulatory (draft) guidelines on DDI by both FDA and European Medicines Agency (EMA) reflect the role of modelling and simulation in DDI studies. Wagner and co-workers evaluated 26 DDI cases with various CYP inhibitions for 15 substrate PBPK models submitted by nine sponsors between 2009 and 2013. They used the predicted mean exposure ratio/observed mean exposure ratio (R_predicted/observed_) as the metric to assess the predictive performance of the PBPK models for maximum plasma concentration (C_max_) or area under the plasma concentration–time curve (AUC) in the presence of CYP inhibition/C_max_ or AUC in the absence of CYP inhibition. In 81 and 77 % of cases, respectively, the R_predicted/observed_ values for AUC and C_max_ ratios were within 1.25-fold of the observed data and, for all the cases, they were within a 2-fold range [38]. In another study, the predictive performance of PBPK models, using a commercial PBPK software, for the effect of CYP3A inducers on 11 substrate PBPK models developed by 6 sponsors was assessed within 13 clinical interaction studies [39]. Using the same metric for assessing the performance of the PBPK model, in 77 and 83 % of the cases, the R_predicted/observed_ values for AUC and C_max_ ratios were within 1.25-fold of the observed data. The regulatory incentives and challenges in conducting and obtaining data in paediatrics have significantly contributed to the increased use of PBPK models in this special population [17, 20].

### Impact of PBPK Modelling on Drug Labels

Since the number of regulatory submissions using PBPK models is rapidly increasing, the regulators have started discussing various aspects of the best practice in PBPK modelling with researchers in the field. Zhao and co-workers have described the best practice in the use of PBPK modelling and simulation for a PBPK regulatory submission to address clinical pharmacology questions and summarised what contents should be included [41].

In 2014, both FDA and EMA ran workshops to discuss current practices and issues in PBPK modelling aiming to improve a common understanding of its utility and limitations, as well as facilitating consensus on best practice in the development, qualification, application and reporting of PBPK modelling activities. These efforts indicate the transition of PBPK from academic curiosity to industrial norm [29]. The reports from both workshops as well as an industry perspective are now published [18, 35, 40].

Arguably, the most important impact of PBPK simulations has been their appearance on the drug labels which is a significant achievement in a relatively short period when they have been used to predict PK properties of pharmaceutical agents. Over the last few years, a number of drug labels are informed by the simulation results generated using PBPK models. These cases show that the simulations either are used in lieu of conducting clinical studies or have informed the drug label that otherwise would have been silent on some specific situations. Table 1 shows an indicative list of approved drugs where PBPK simulations have informed the drug label.

**Table 1:** An indicative list of approved drug where PBPK simulations have informed the drug label (FDA, EMA and PMDA)

Number	Company	Active ingredient	Drug name	Clinical pharmacology and biopharmaceutic review/drug label
1	Pfizer	Sildenafil citrate	Revatio	http://www.accessdata.fda.gov/drugsatfda_docs/nda/2009/022473s000_ClinPharmR.pdf
				http://www.accessdata.fda.gov/drugsatfda_docs/label/2012/022473s003lbl.pdf
2	Janssen	Rivaroxaban	Xarelto	http://www.accessdata.fda.gov/drugsatfda_docs/nda/2011/022406Orig1s000ClinPharmR.pdf
				http://www.accessdata.fda.gov/drugsatfda_docs/label/2011/022406s000lbl.pdf
3	Ariad	Ponatinib hydrochloride	Iclusig	http://www.accessdata.fda.gov/drugsatfda_docs/nda/2012/203469Orig1s000ClinPharmR.pdf
				http://www.accessdata.fda.gov/drugsatfda_docs/label/2012/203469lbl.pdf
4	Janssen	Simeprevir sodium	Olysio	http://www.accessdata.fda.gov/drugsatfda_docs/nda/2013/205123Orig1s000ClinPharmR.pdf
				http://www.accessdata.fda.gov/drugsatfda_docs/label/2013/205123s001lbl.pdf
5	Pharmacyclics	Ibrutinib	Imbruvica	http://www.accessdata.fda.gov/drugsatfda_docs/nda/2013/205552Orig1s000ClinPharmR.pdf
				http://www.accessdata.fda.gov/drugsatfda_docs/label/2013/205552s000lbl.pdf
				http://www.ema.europa.eu/docs/en_GB/document_library/EPAR_-_Product_Information/human/003791/WC500177775.pdf (EMA)
6	Actelion	Macitentan	Opsumit	http://www.accessdata.fda.gov/drugsatfda_docs/nda/2013/204410Orig1s000ClinPharmR.pdf
				http://www.accessdata.fda.gov/drugsatfda_docs/label/2013/204410s000lbl.pdf
				http://www.ema.europa.eu/docs/en_GB/document_library/EPAR_-_Product_Information/human/002697/WC500160899.pdf (EMA)
7	Astrazeneca	Naloxegol oxalate	Movantik	http://www.accessdata.fda.gov/drugsatfda_docs/nda/2014/204760Orig1s000ClinPharm.pdf
				http://www.accessdata.fda.gov/drugsatfda_docs/label/2014/204760s000lbl.pdf
8	Genzyme	Eliglustat tartrate	Cerdelga	http://www.accessdata.fda.gov/drugsatfda_docs/nda/2014/205494Orig1s000ClinPharmR.pdf
				http://www.accessdata.fda.gov/drugsatfda_docs/label/2014/205494Orig1s000lbl.pdf
				http://www.info.pmda.go.jp/downfiles/ph/PDF/340531_3999037M1023_1_02.pdf (PMDA)
				http://www.ema.europa.eu/docs/en_GB/document_library/EPAR_-_Product_Information/human/003724/WC500182387.pdf (EMA)
9	Incyte	Ruxolitinib phosphate	Jakafi	http://www.accessdata.fda.gov/drugsatfda_docs/nda/2011/202192Orig1s000PharmR.pdf
				http://www.accessdata.fda.gov/drugsatfda_docs/label/2014/202192s006lbl.pdf
10	Novartis	Ceritinib	Zykadia	http://www.accessdata.fda.gov/drugsatfda_docs/nda/2014/205755Orig1s000ClinPharmR.pdf
				http://www.accessdata.fda.gov/drugsatfda_docs/label/2014/205755s000lbl.pdf
				http://www.ema.europa.eu/docs/en_GB/document_library/EPAR_-_Product_Information/human/003819/WC500187504.pdf (EMA)
11	Astrazeneca	Olaparib	Lynparza	http://www.accessdata.fda.gov/drugsatfda_docs/nda/2014/206162Orig1s000ClinPharmR.pdf
				http://www.accessdata.fda.gov/drugsatfda_docs/label/2014/206162lbl.pdf
12	Eisai	Lenvatinib mesylate	Lenvima	http://www.accessdata.fda.gov/drugsatfda_docs/nda/2015/206947Orig1s000ClinPharmR.pdf
				http://www.accessdata.fda.gov/drugsatfda_docs/label/2015/206947s000lbl.pdf
13	Novartis	Panobinostat lactate	Farydak	http://www.accessdata.fda.gov/drugsatfda_docs/nda/2015/205353Orig1s000ClinPharmR.pdf
				http://www.accessdata.fda.gov/drugsatfda_docs/label/2015/205353s000lbl.pdf
				http://www.pmda.go.jp/PmdaSearch/iyakuDetail/ResultDataSetPDF/300242_4291040M1023_1_01 (PMDA)
14	Janssen	Rilpivirine hydrochloride	Edurant	http://www.accessdata.fda.gov/drugsatfda_docs/nda/2011/202022Orig1s000ClinPharmR.pdf
				http://www.accessdata.fda.gov/drugsatfda_docs/label/2011/202022s000lbl.pdf
15	Alkermes	Aripiprazole lauroxil	Aristada	http://www.accessdata.fda.gov/drugsatfda_docs/nda/2015/207533Orig1s000ClinPharmR.pdf
				http://www.accessdata.fda.gov/drugsatfda_docs/label/2015/207533s000lbl.pdf
16	Genentech	Cobimetinib fumarate	Cotellic	http://www.accessdata.fda.gov/drugsatfda_docs/nda/2015/206192Orig1s000ClinPharmR.pdf
				http://www.accessdata.fda.gov/drugsatfda_docs/label/2015/206192s000lbl.pdf
17	Novartis	Sonidegib phosphate	Odomzo	http://www.accessdata.fda.gov/drugsatfda_docs/nda/2015/205266Orig1s000ClinPharmR.pdf
				http://www.accessdata.fda.gov/drugsatfda_docs/label/2015/205266s000lbl.pdf
				http://www.ema.europa.eu/docs/en_GB/document_library/EPAR_-_Product_Information/human/002839/WC500192970.pdf (EMA)
18	Genentech	Alectinib hydrochloride	Alecensa	http://www.accessdata.fda.gov/drugsatfda_docs/nda/2015/208434Orig1s000ClinPharmR.pdf
				http://www.accessdata.fda.gov/drugsatfda_docs/label/2015/208434s000lbl.pdf
19	Astrazeneca	Osimertinib mesylate	Tagrisso	http://www.accessdata.fda.gov/drugsatfda_docs/nda/2015/208065Orig1s000ClinPharmR.pdf
				http://www.accessdata.fda.gov/drugsatfda_docs/label/2015/208065s000lbl.pdf

For detailed information about specific drug products, visit the FDA-approved drug product database (http://www.accessdata.fda.gov/scripts/cder/drugsatfda) or the relevant regulatory agency website

### Conclusions and Future Directions

PBPK models using nonclinical and clinical data to predict drug PK/PD properties in healthy and patient subjects are increasingly used at various stages of drug development and regulatory interactions. However, initially the regulatory applications of PBPK models were mainly focused on predicting DDI, the areas of application are gradually expanding. These are expanded in other areas such as drug formulation and/or absorption modelling, age- and ethnic-related changes in PK and disposition (e.g. paediatrics/geriatrics and Japanese/Chinese populations) and the assessment of PK changes in case of different physiopathological conditions (e.g. renal and/or hepatic deficiencies) [32].

Undoubtedly, current PBPK models are far from perfect, and as their applications grow, our knowledge of their abilities and weaknesses improves. Perhaps, the biggest challenge in further expansion of PBPK models is the lack of adequate and reliable systems data. As the models become more complex, they demand more detailed knowledge of the system, and while there has been significant improvement in identifying and generating missing information, we still have a long way to go. Systems data, such as the abundance and activity of non-CYP enzymes and transporters in various tissues, absorption-related data and how these are changing by age/disease status, are generally lacking. Such challenges are even bigger when developing mechanistic PBPK models for biologics and/or models that aim at providing mechanistic insights into drug efficacy and safety. However, since these data are related to the biological system, the burden and benefit of generating these data can be shared through collaboration between various stakeholders in a pre-competitive manner. The value of collaborative efforts to address key public health regulatory science issues is highlighted in [22].

Over the last two decades, we have been able to improve in vitro experiments and develop techniques to extrapolate to the in vivo situation [12]. As the in vitro experiments become more complex, it is necessary to apply advanced modelling techniques to the in vitro data to extract detailed and accurate data to improve the predictive performance of PBPK models.

Developing bottom-up system pharmacology models requires inputs from numerous experts including experimentalists, biologists, epidemiologists, pharmacists, pharmacologists and mathematical modellers. Therefore, by its nature, it is a multi-disciplinary endeavour requiring adequate education and communication among various stakeholders who have traditionally been working in isolation. These experts usually work independent of each other and need to learn how to interact and communicate with other disciples. Furthermore, the best practice in developing and accessing such models are evolving. While there are few articles in literature that provide guidelines, more rigorous standards are lacking as highlighted in [32]. Regulatory agencies around the world have started addressing these needs and providing guidelines. A PBPK concept paper has been published by EMA which may lead to a specific European guideline on qualification and reporting of PBPK modelling and analysis (EMA/CHMP 2014).

The applications of PBPK models will expand even further if they are adequately equipped to connect and interact with other tools/platforms (interoperability), such as quantitative system pharmacology models of various disease progressions. Innovative applications of PBPK have started appearing in the literature, e.g. introducing time-varying physiology into paediatric PBPK models [1], modelling drug disposing in kidney [13], brain [8] and lung [9], virtual bioequivalent studies [6], modelling antibody drug conjugates (ADC) [4], etc. In addition, given the integrative nature of PBPK models, which allows incorporating characteristics of patients, it will not be surprising to see applications of these models in implementing precision dosing at the point of care in the near future. In particular, when relevant and affordable biomarkers for enzyme/transporters activities are developed/identified, such information can be incorporated within PBPK models to determine and optimise doses in e.g. DDI cases.
